# Bearing-Only Obstacle Avoidance Based on Unknown Input Observer and Angle-Dependent Artificial Potential Field

**DOI:** 10.3390/s19010031

**Published:** 2018-12-21

**Authors:** Xiaohua Wang, Yan Liang, Shun Liu, Linfeng Xu

**Affiliations:** School of Automation, Northwestern Polytechnical University, Xi’an 710072, China; xiaohuawang311@sina.com (X.W.); liushun_ls@yeah.net or liushun_ls@mail.nwpu.edu.cn (S.L.)

**Keywords:** path planning, obstacle avoidance, unknown input observer

## Abstract

This paper presents the problem of obstacle avoidance with bearing-only measurements in the case that the obstacle motion is model-free, i.e., its acceleration is absolutely unknown, which cannot be dealt with by the mainstream Kalman-like schemes based on the known motion model. First, the essential reason of the collision caused by local minimum problem in the standard artificial potential field method is proved, and hence a revised method with angle dependent factor is proposed. Then, an unknown input observer is proposed to estimate the position and velocity of the obstacle. Finally, the numeric simulation demonstrates the effectiveness in terms of estimation accuracy and terminative time.

## 1. Introduction

To travel safely in unstructured environments, it is crucial for agents to be able to plan their paths adaptively and optimally, even in the absence of a priori knowledge. This task is referred to as obstacle avoidance (OA) [1,2].

One important issue of OA is to design a collision-avoidance path given positions and velocities of obstacles. Artificial Potential Field (APF) method [3] is one of the most popular planners. APF and its variants [4,5,6] are cost-effective but face the local minimum problem, i.e., the agent is trapped at the local minimum position and hence collides with obstacles or loses the possibility of reaching the destination. Although much attention has been paid to the method revision (e.g., [7,8,9]), the existence condition of the collision caused by local minimum (LM) has not been explored and hence these revision schemes are somewhat ad hoc. The corresponding existence condition needs to be found and furthermore a suitable revision needs to be presented.

Another important issue and precondition of OA is to estimate obstacles’ states, such as positions and velocities, precisely. Given the a priori movement model of obstacles, the Kalman filter or its green variants [10,11,12,13,14] yelloware utilized to estimate states of obstacles. According to the basic principles, these methods can be divided into three classes. The first one is multi-model matching [15,16], where a set of models is designed to cover the possible obstacle motions and the stochastic model switch is considered as a Markov chain [17]. The second scheme is clustering-based [18,19], where the previously-obtained obstacle trajectories are classified into multiple clusters for matching the current obstacle track. The third one is Gaussian-mixture approximation [20,21,22], where the means and covariances of Gaussian motion models are adaptively self-learning. In general, all these schemes are computation-intensive due to multi-model filtering, multi-hypothesis mode recognition, or multi-parameter learning. Moreover, it is important to mention that these schemes absolutely depend on a priori models and hence will definitely become invalid in the incomplete mode/hypothesis/parameter set case, which may exist in unstructured or even hostile environment.

Actually, such model-unknown cases amount to the dynamic equations with unknown transition matrix and unknown input. Moreover, a bearing measurement corresponds to infinite position solutions. As a result, it is unlikely to derive accurate state using the traditional filtering algorithms. Although the unknown input observer (UIO) was utilized to reconstruct the states for dynamic systems with unknown input in the automation field, it would be invalid for the model-unknown case with bearing-only measurements since a known transition matrix is necessary for the UIO calculation. It is highly demanded to develop a cost-effective method to reconstruct states for the model unknown motions with bearing-only observations.

In this paper, an OA scheme with obstacle bearing-only measurement is proposed for the case that the obstacle motion model is general. One contribution is to explore a sufficient collision condition of the APF, and further propose the revised APF. The other is to design an unknown input observer to estimate obstacles’ states, which avoids the precondition that the motion model should not contain unknown parameters or input required by the mainstream Kalman-like schemes. The proposed unknown input observer also has potential applications in the field of tracking and clustering.

Throughout this paper, the symbols · and × represents dot product and cross product, respectively; the subscript ⊥ represents the perpendicular operation of the vector; and I denotes the identity matrix with proper dimensions.

## 2. Problem Formulation

Consider the OA task with an agent and multiple obstacles, all mass-points in the two-dimensional Cartesian X-Y coordinates. The acceleration of the *i*th obstacle at time *t* is

(1) $${\ddot{p}}_{O_{i}}\left(t\right)=f_{i}\left(t\right)$$

where $f_{i}\left(t\right)$ is an unknown time-varying function, representing arbitrary possible movement or unexpectable maneuver. In other words, Equation (1) means that the motion is general without special requirement or a priori movement information, i.e., model-free. The acceleration of the agent at position $p_{A}\left(t\right)$ is

(2) $${\ddot{p}}_{A}\left(t\right)=f_{A}\left(t\right)$$

where $f_{A}\left(t\right)$ is determined according to the task of path planning with the constraint that $∥f_{A}\left(t\right)∥\leq \;a_{max}$, i.e., the maximum acceleration is given. Besides, the position and the velocity of the agent, $p_{A}\left(t\right)$ and ${\dot{p}}_{A}\left(t\right)$, are both known.

Here, the object of path planning should satisfy two requirements. One is minimum safety distance, i.e.,

(3) $$\min_{f_{A}}∥p_{A}\left(t\right)-p_{O_{i}}\left(t\right)∥\geq \delta_{1},\;\forall t$$

where $\delta_{1}>0$ represents the permissible minimum safe distance. The other is destination reachability, i.e.,

(4) $$∥p_{A}\left(t_{f}\right)-p_{des}∥\leq \delta_{2},\exists t_{f}<\infty$$

where $p_{des}$ is the destination position and $\delta_{2}\geq 0$ is the permissible maximum navigation error.

**Remark** **1.** *To satisfy these two requirements, the OA task has to be divided into two subtasks. One is to design a path planner to generate a path automatically to reach the destination under the constraint of the minimum safety distance. The other is to to estimate $p_{O_{i}}\left(t\right)$ and ${\dot{p}}_{O_{i}}\left(t\right)$ from bearing measurements to guarantee the first requirement:*

- *In planner design, artificial potential field method is cost-effective, but may involved in the LM and hence collide with obstacles in some cases. In other words, it is highly demanded to explore the collision condition of the APF, and further present the reasonable revision to avoid the local minimum.*
- *In obstacle state estimation, the traditional Kalman-like state estimators, whose applicability or optimality depends on the a priori model, will hence become inapplicable in the presence of unknown acceleration as the unknown model parameters or unexpected model mismatch.*

## 3. Angle-Dependent APF

The acceleration of the agent can be written as

(5) $$f_{A}\left(t\right)=\left\{\begin{array}{cc} F(t) & \mathrm{if}\;∥F\left(t\right)∥\leq a_{max}\\ a_{max}\frac{F(t)}{∥F(t)∥} & \mathrm{otherwise} \end{array}\right.$$

where F(t) is the force generates by a path planner given the $p_{O_{i}}\left(t\right)$ and ${\dot{p}}_{O_{i}}\left(t\right)$.

The resultant of forces generated by APF is

(6) $$F\left(t\right)=F_{att}\left(t\right)+F_{rep1}\left(t\right)+F_{rep2}\left(t\right)$$

where

(7) $$F_{att}\left(t\right)=\alpha_{p}{∥p_{des}-p_{A}\left(t\right)∥}^{m}n_{AD}$$

(8) $$F_{rep1}\left(t\right)=\sum_{i}\frac{-\eta (2a_{max}+v_{AO_{i}}\left(t\right))}{2a_{max}{\left(\rho_{i}\left(t\right)-\rho_{m}\left(t\right)\right)}^{2}}\varphi_{i}\left(t\right)$$

(9) $$F_{rep2}=\sum_{i}\frac{-\eta v_{AO_{i}}\left(t\right)v_{AO_{i}⊥}\left(t\right)}{a_{max}\rho_{i}\left(t\right){\left(\rho_{i}\left(t\right)-\rho_{m_{i}}\left(t\right)\right)}^{2}}\varphi_{i⊥}\left(t\right)$$

$\varphi_{i}\left(t\right)=\frac{p_{O_{i}}\left(t\right)-p_{A}\left(t\right)}{∥p_{O_{i}}\left(t\right)-p_{A}\left(t\right)∥}$, $v_{AO_{i}}\left(t\right)={\left[v_{A}\left(t\right)-v_{O_{i}}\left(t\right)\right]}^{T}\varphi_{i}\left(t\right)$, $\rho_{i}\left(t\right)=∥p_{A}\left(t\right)-p_{O_{i}}\left(t\right)∥$, $\rho_{m_{i}}\left(t\right)=\frac{v_{AO_{i}}^{2}\left(t\right)}{2a_{max}}$, $v_{A}\left(t\right)={\dot{p}}_{A}\left(t\right)$ and $v_{O_{i}}\left(t\right)={\dot{p}}_{O_{i}}\left(t\right)$. $\alpha_{p},m$ and η are positive constants and $n_{AD}$ is the unit direction vector from the agent to the destination.

**Theorem 1.**  *(Collision Condition) Considering one obstacle case in the artificial potential field method in Equations *(7)*–*(9)*, and assuming that backward movement is not allowed for agent, $\exists 0<t_{o}<\infty$, there holds $p_{O_{i}}\left(t_{o}\right)=p_{A}\left(t_{o}\right)$, if*

(10) $$\frac{\left(v_{A}\left(t\right)-v_{O_{i}}\left(t\right)\right)\cdot \left(p_{O_{i}}\left(t\right)-p_{A}\left(t\right)\right)}{|v_{A}\left(t\right)-v_{O_{i}}\left(t\right)||p_{O_{i}}\left(t\right)-p_{A}\left(t\right)|}=1,t>0$$

*and*

(11) $$|\left(p_{O_{i}}\left(t\right)-p_{A}\left(t\right)\right)\times \left(p_{O_{i}}\left(t\right)-p_{des}\right)|=0,t>0$$

**Proof** **of Theorem 1.** The equation of the $F_{rep2}$ for one obstacle is

(12) $$F_{rep2}=\frac{-\eta v_{AO_{i}}\left(t\right)v_{AO_{i}⊥}\left(t\right)}{a_{max}\rho_{i}\left(t\right){\left(\hat{\rho_{i}}\left(t\right)-\rho_{m_{i}}\left(t\right)\right)}^{2}}\varphi_{i⊥}\left(t\right)$$

where $v_{AO_{i}⊥}=\sqrt{∥v_{A}\left(t\right)-v_{O_{i}}{\left(t\right)∥}^{2}-v_{AO_{i}}^{2}\left(t\right)}$. Since $v_{A}\left(t\right)={\left[v_{ax},v_{ay}\right]}^{T}$, $p_{A}\left(t\right)={\left[x_{a},y_{a}\right]}^{T}$, $v_{O_{i}}\left(t\right)={\left[v_{oxi},v_{oyi}\right]}^{T}$ and $p_{O_{i}}\left(t\right)={\left[x_{oi},y_{oi}\right]}^{T}$, we have

(13) $$\begin{array}{cc} v_{AO_{i}⊥} & =\sqrt{∥{\left[v_{ax},v_{ay}\right]}^{T}-{\left[v_{oxi},v_{oyi}\right]}^{T}∥-v_{AO_{i}}^{2}\left(t\right)}\\ & =\sqrt{A^{2}+B^{2}-{\left(\frac{\left[A,B\right]{\left[C,D\right]}^{T}}{\sqrt{{\left(C\right)}^{2}+{\left(D\right)}^{2}}}\right)}^{2}}\\ & =\sqrt{B^{2}C^{2}+A^{2}D^{2}-2ABCD}\\ & =\sqrt{{\left(BC-AD\right)}^{2}} \end{array}$$

where $A=v_{ax}-v_{oxi},B=v_{ay}-v_{oyi},C=x_{oi}-x_{a}$, and $D=y_{oi}-y_{a}$. The condition in Equation (9) represents the case that $\left(v_{A}\left(t\right)-v_{O_{i}}\left(t\right)\right)$ and $\left(p_{O_{i}}\left(t\right)-p_{A}\left(t\right)\right)$ have the same direction, i.e., BC=AD. Then, we have $v_{AO_{i}⊥}=0$ and $F_{rep2}=0$ from Equations (13) and (12). According to Equation (11), $F_{rep1}$ and $F_{att}\left(t\right)$ are collinear. Finally, F(t) and $F_{att}\left(t\right)$ are collinear, i.e., the agent either moves along the obstacle-agent line or remains stationary. Because the obstacle is moving toward the agent and the agent does not permit the backward movement, one collision will definitely happen, i.e., $∥p_{O_{i}}\left(t\right)-p_{A}\left(t\right)∥=0$ will hold at a finite time *t*. ☐

**Figure 1:**
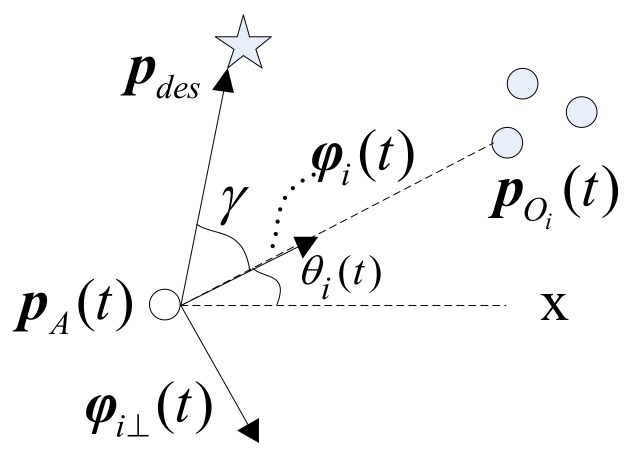
The illustration of relationship between variables.

As shown in Figure 1, γ denotes the relative angle of two lines: one of which crosses through the agent and the obstacle; another crosses through the agent and the destination. Obviously, γ=0 is equal to Equation (11). Theorem 1 implies that the collision will be inevitable if $F_{rep2}=0$ under condition Equations (10) and (11). In other words, we should make $F_{rep2}$ non-zero in the case of γ=0, which can be implemented through introducing an angle-dependent factor β(γ) to Equation (12),

(14) $$F_{rep2}=\frac{-\eta v_{AO_{i}}\left(t\right)\left(v_{AO_{i}⊥}\left(t\right)+\alpha \beta \left(\gamma \right)\right)}{a_{max}\rho_{i}\left(t\right){\left(\hat{\rho_{i}}\left(t\right)-\rho_{m_{i}}\left(t\right)\right)}^{2}}\varphi_{i⊥}\left(t\right)$$

to guarantee $F_{rep2}\neq 0$ under Equations (10) and (11). In Equation (14), a positive constant α represents a given weight to control the effect of β. We suggest one possible choice: $\beta \left(\gamma \right)=n_{AD}\varphi_{i}\left(t\right)=\cos\left(\gamma \right)$. Then, Equation (9) can be rewrite as:

(15) $$F_{rep2}=\sum_{i}\frac{-\eta v_{AO_{i}}\left(t\right)\left(v_{AO_{i}⊥}\left(t\right)+\alpha \beta \left(\gamma \right)\right)}{a_{max}\rho_{i}\left(t\right){\left(\hat{\rho_{i}}\left(t\right)-\rho_{m_{i}}\left(t\right)\right)}^{2}}\varphi_{i⊥}\left(t\right)$$

## 4. Unknown Input Observer for Bearing-Only Tracking

In Section 3, states as positions and velocities of obstacles are all known information for the planner, while in practice they often need to be determined from sensor data. Considering that the obstacle motion is general, i.e., acceleration is the unknown input (UI), traditional Kalman-like filter and observer are all invalid for determining positions and velocities of obstacles, because they both rely on the system state without UI. Thus, the problem boils down to the the unknown input observer (UIO) design. The existing UIO has been applied in the field of fault detection, in the linear model through decoupling UI estimate error with UI [23]. However, reconstructing position and velocity from bearing is the distinct and open non-linear estimation problem in the presence of UI. In this section, a non-linear UIO is proposed to estimate positions and velocities of obstacles, which fill up the blank of the field.

As shown in Figure 1, $\theta_{i}\left(t\right)$ is the bearing-only measurement obtained by a sensor, for example monocular camera [24]. Then, a direction unit vector (DUV), which is also needed in Equation (8), is denoted as $\varphi_{i}\left(t\right)$ along the direction of the vector $p_{O_{i}}\left(t\right)-p_{A}\left(t\right)$ with respective to the X axis coordinate:

(16) $$\varphi_{i}\left(t\right)=\frac{p_{O_{i}}\left(t\right)-p_{A}\left(t\right)}{∥p_{O_{i}}\left(t\right)-p_{A}\left(t\right)∥}={\left[\cos\theta_{i}\left(t\right)\;\sin\theta_{i}\left(t\right)\right]}^{T}$$

where $\theta_{i}\left(t\right)$ is the angle.

### 4.1. Position Estimation

**Figure 2:**
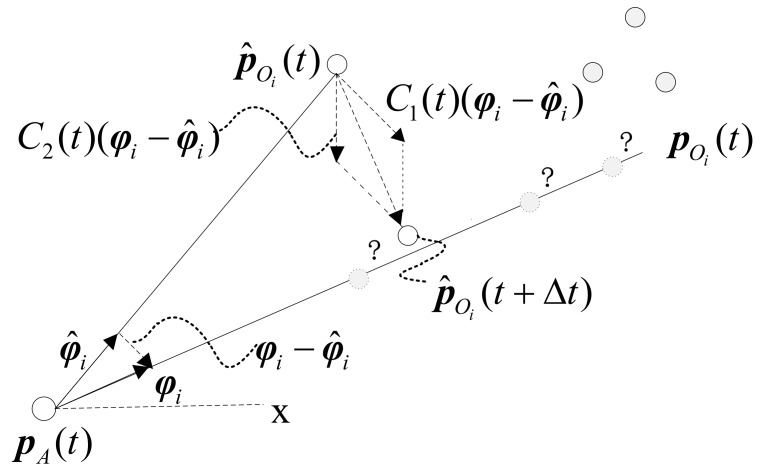
The principle of position estimation of our UIO.

As shown in Figure 2, an agent is at $p_{A}\left(t\right)$; the DUV of *i*th obstacle is denoted by $\varphi_{i}\left(t\right)$. Question marks represent the situation that the true obstacle position $p_{O_{i}}\left(t\right)$ is unknown. The DUV of ${\hat{p}}_{O_{i}}\left(t\right)$ is ${\hat{\varphi }}_{i}\left(t\right)=\frac{{\hat{p}}_{O_{i}}\left(t\right)-p_{A}\left(t\right)}{∥{\hat{p}}_{O_{i}}\left(t\right)-p_{A}\left(t\right)∥}$.

Generally speaking, the design idea of our UIO estimator is to make the estimate close to the true state along the relative direction. In other words, if ${\hat{\varphi }}_{i}\left(t\right)$ is not as same as $\varphi_{i}\left(t\right)$, then ${\hat{p}}_{O_{i}}\left(t\right)$ should be modified to be close to the line $p_{O_{i}}\left(t\right)-p_{A}\left(t\right)$. Specifically, the estimate should satisfy the following conditions.

① If the DUV of the true position equals the DUV of the estimate, then no modification is needed.
② The direction of the estimate should point to the line $p_{O_{i}}\left(t\right)-p_{A}\left(t\right)$.

Denote

(17) $${\dot{\hat{p}}}_{O_{i}}\left(t\right)=\lim_{▵t\to 0}\frac{{\hat{p}}_{O_{i}}\left(t+▵t\right)-{\hat{p}}_{O_{i}}\left(t\right)}{▵t}$$

then one possible scheme for position estimation is

(18) $${\dot{\hat{p}}}_{O_{i}}\left(t\right)=C\left(t\right)\left(\varphi_{i}\left(t\right)-{\hat{\varphi }}_{i}\left(t\right)\right)$$

where $C\left(t\right)=C_{1}\left(t\right)+C_{2}\left(t\right)$, $C_{1}\left(t\right)=k_{1}\left(I-{\hat{\varphi }}_{i}\left(t\right)\varphi_{i}{\left(t\right)}^{T}\right)$, $C_{2}\left(t\right)=k_{2}\left(1-{\hat{\varphi }}_{i}{\left(t\right)}^{T}\varphi_{i}\left(t\right)\right)$, while $k_{1}$ and $k_{2}$ are positive numbers.

Recalling two conditions, we can check the above conditions as follows:

① If the bearing of the true position equals the bearing of the estimate, i.e., $\varphi_{i}\left(t\right)={\hat{\varphi }}_{i}\left(t\right)$. We have both C(t)=0 and $\varphi_{i}\left(t\right)-{\hat{\varphi }}_{i}\left(t\right)=0$ and hence ${\dot{\hat{p}}}_{O_{i}}\left(t\right)=0$.
② Obviously, $C_{2}\left(t\right)$ is scalar and hence $C_{2}\left(t\right)\left(\varphi_{i}\left(t\right)-{\hat{\varphi }}_{i}\left(t\right)\right)$ remains the direction of $\left(\varphi_{i}\left(t\right)-{\hat{\varphi }}_{i}\left(t\right)\right)$.

### 4.2. Velocity Estimation of UIO

From Equation (16), we have

(19) $$\begin{array}{c} \varphi_{i}\left(t+▵t\right)-\varphi_{i}\left(t\right)\\ =\frac{1}{∥p_{O_{i}}\left(t+▵t\right)-p_{A}\left(t+▵t\right)∥}(p_{O_{i}}\left(t+▵t\right)\\ \;\;\;-p_{O_{i}}\left(t\right)-\left(p_{A}\left(t+▵t\right)-p_{A}\left(t\right)\right)\\ \;\;\;+\left(1-\frac{\left(∥p_{O_{i}}\left(t+▵t\right)-p_{A}\left(t+▵t\right)∥\right)}{∥p_{O_{i}}\left(t\right)-p_{A}\left(t\right)∥}\right)\left(p_{O_{i}}\left(t\right)-p_{A}\left(t\right)\right)) \end{array}$$

Furthermore,

(20) $$\begin{array}{c} ∥p_{O_{i}}\left(t+▵t\right)-p_{A}\left(t+▵t\right)∥\left(\varphi_{i}\left(t+▵t\right)-\varphi_{i}\left(t\right)\right)\\ -\left(1-\frac{\left(∥p_{O_{i}}\left(t+▵t\right)-p_{A}\left(t+▵t\right)∥\right)}{∥p_{O_{i}}\left(t\right)-p_{A}\left(t\right)∥}\right)\left(p_{O_{i}}\left(t\right)-p_{A}\left(t\right)\right)\\ =p_{O_{i}}\left(t+▵t\right)-p_{O_{i}}\left(t\right)-\left(p_{A}\left(t+▵t\right)-p_{A}\left(t\right)\right) \end{array}$$

and hence

(21) $$\begin{array}{c} ∥p_{O_{i}}\left(t+▵t\right)-p_{A}\left(t+▵t\right)∥\varphi_{i}\left(t+▵t\right)\\ -∥p_{O_{i}}\left(t\right)-p_{A}\left(t\right)∥\varphi_{i}\left(t\right))\\ =p_{O_{i}}\left(t+▵t\right)-p_{O_{i}}\left(t\right)-\left(p_{A}\left(t+▵t\right)-p_{A}\left(t\right)\right). \end{array}$$

Through dividing *t* on both sides of Equation (19) and taking the limit ▵t⇀0, we have

(22) $$\;{\dot{\varphi }}_{i}\left(t\right)=\lim_{▵t\to 0}\frac{\varphi_{i}\left(t+▵t\right)-\varphi_{i}\left(t\right)}{▵t}=\frac{{\dot{p}}_{O_{i}}\left(t\right)-{\dot{p}}_{A}\left(t\right)}{∥p_{O_{i}}\left(t\right)-p_{A}\left(t\right)∥}$$

or

(23) $${\dot{p}}_{O_{i}}\left(t\right)={\dot{p}}_{A}\left(t\right)+∥p_{O_{i}}\left(t\right)-p_{A}\left(t\right)∥{\dot{\varphi }}_{i}\left(t\right)$$

which can be treated as the constraint between ${\dot{p}}_{O_{i}}\left(t\right)$ and $p_{O_{i}}\left(t\right)$. Because it is expected to obtain ${\hat{\dot{p}}}_{O_{i}}\left(t\right)$ as near as possible to ${\dot{p}}_{O_{i}}\left(t\right)$, such constraint is also accepted in constructing the UIO:

(24) $${\hat{\dot{p}}}_{O_{i}}\left(t\right)={\dot{p}}_{A}\left(t\right)+∥{\hat{p}}_{O_{i}}\left(t\right)-p_{A}\left(t\right)∥{\dot{\varphi }}_{i}\left(t\right)$$

Here, Equation (24) is obtained from Equation (23) by substituting $p_{O_{i}}\left(t\right)$ with ${\hat{p}}_{O_{i}}\left(t\right)$. To decrease the effect of improper initialization, a discount factor 0<d(t)<1 is introduced for UIO initialization

(25) $${\hat{\dot{p}}}_{O_{i}}\left(t\right)={\dot{p}}_{A}\left(t\right)+d\left(t\right)∥{\hat{p}}_{O_{i}}\left(t\right)-p_{A}\left(t\right)∥{\dot{\varphi }}_{i}\left(t\right)$$

for $t\leq t_{in}$ where $t_{in}$ is initialization terminate instant.

**Figure 3:**
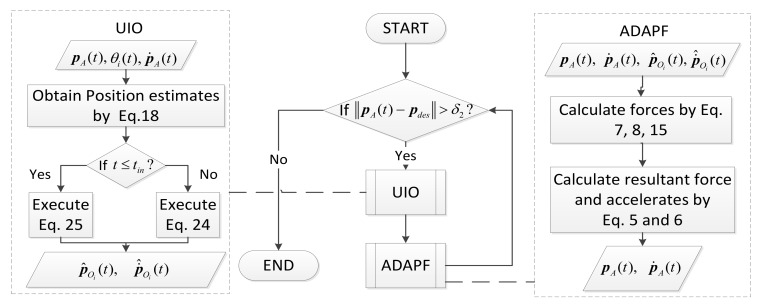
The flowchart of the algorithm.

The total method with its flowchart is shown in Figure 3.

## 5. Simulation and Analysis

Three scenarios to test the performance of our method were considered. The first scenario was set to test the performance of UIO estimator, which includes one serpentine motion agent and one obstacle has “curve-maneuver” or “constant-velocity”, respectively. To demonstrate validation of our path planner with the ability of escaping the local minimum point, the second scenario considered the situation that one agent and one obstacle move head on given the position of the obstacle. The third scenario contained multiple moving and stationary obstacles to test the integrated performance of obstacle estimation and path planner. In general, $∥{\hat{p}}_{O_{i}}\left(t\right)-p_{A}\left(t\right)∥$ is much larger than the amplitude of ${\hat{\dot{p}}}_{O_{i}}\left(t\right)$ at the beginning, so the factor d(t) should be small, e.g., d(t)=0.01 in Equation (25). Based on the velocity of the agent, $k_{1}$ and $k_{2}$ were set to 5 for agent’s velocity ${\left[5+10sin\left(0.02t\right),5\right]}^{T}$ m/s in Scenario 1 and 10 for agent’s velocity ${\left[10a,10b\right]}^{T}m/s\;\left(a,b\in \left\{1,-1\right\}\right)$ in Scenario 3, respectively. In Scenarios 2 and 3, the parameters of our path planner were chosen as $\alpha_{p}=0.009$, $\alpha_{\beta }=200$ and η=700; the maximum acceleration was set as $a_{max}=5\;{m/s}^{2}$; from $D_{safe}=v_{A}^{max}+\rho_{0}+3\delta$, the safe distance was set as 31.5 m where δ=0.5 and $v_{A}^{max}=20$.

### 5.1. UIO Estimator

**Figure 4:**
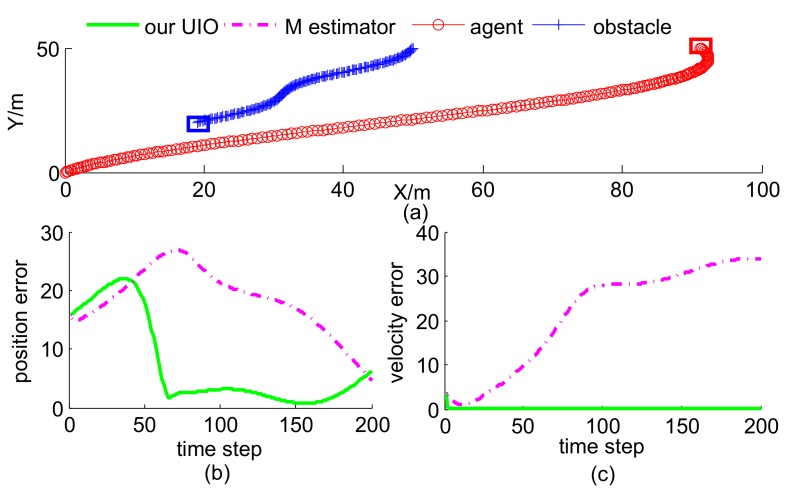
Trajectories and estimation error of Case 1: (**a**) curve-maneuver; (**b**) position error; (**c**) position error.

**Figure 5:**
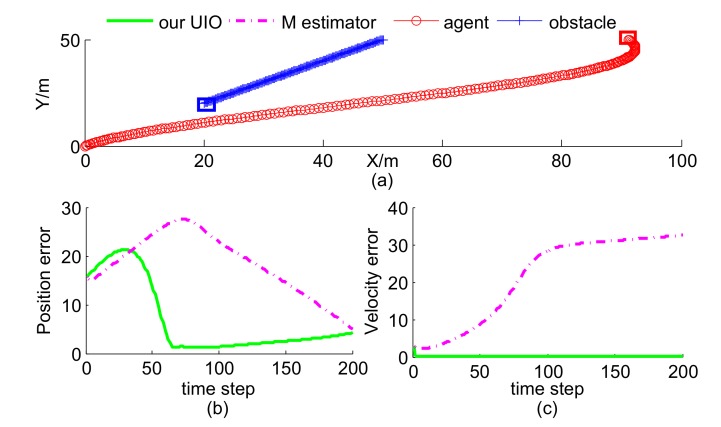
Trajectories and estimation error of Case 2: (**a**) constant-velocity; (**b**) position error; (**c**) position error.

In this scenario, the agent’s trajectory was given, and the obstacle motion contained two cases. Case 1 is curve-maneuver with velocity ${\left[-3+2sin\left(0.05t\right),-3\right]}^{T}m/s$ and Case 2 is constant-velocity with velocity ${\left[-3,-3\right]}^{T}m/s$. The initial position of the obstacle was ${\left[50,50\right]}^{T}m$, whose corresponding estimate was ${\left[65,55\right]}^{T}m$. There is no existing method to solve the same problem as our UIO estimator, so we take M estimator [25] as the rival, which has a similar form as our UIO estimator. It is worth mentioning that M estimator does not obtain velocity estimate, thus we computed the differential of position estimate as its velocity estimate. As shown in Figure 4 and Figure 5, our proposed UIO estimator is much better than M estimator in estimating the position and velocity of the obstacle. Blue and red squares represent terminate positions of obstacle and agent, respectively.

### 5.2. ADAPF

In this scenario, we compared the path planner with APF on minimum distance (MD) between the agent and the obstacle. At the beginning, besides that the motion of the obstacle was opposite the agent motion, the obstacle, the agent and the destination were on the same line, which means the spatial layout satisfies collision condition in Theorem 1. The initial position and velocity of the obstacle were ${\left[170,170\right]}^{T}m$ and ${\left[-5,-5\right]}^{T}m/s$, respectively. The initial position and velocity of the agent were ${\left[0,0\right]}^{T}m$ and ${\left[5,5\right]}^{T}m/s$, respectively. Then, as the initial position changes,γ changes from $0^{\circ }$ to $90^{\circ }$, accordingly. The initial velocity of the agent has the direction pointing to the obstacle.

**Figure 6:**
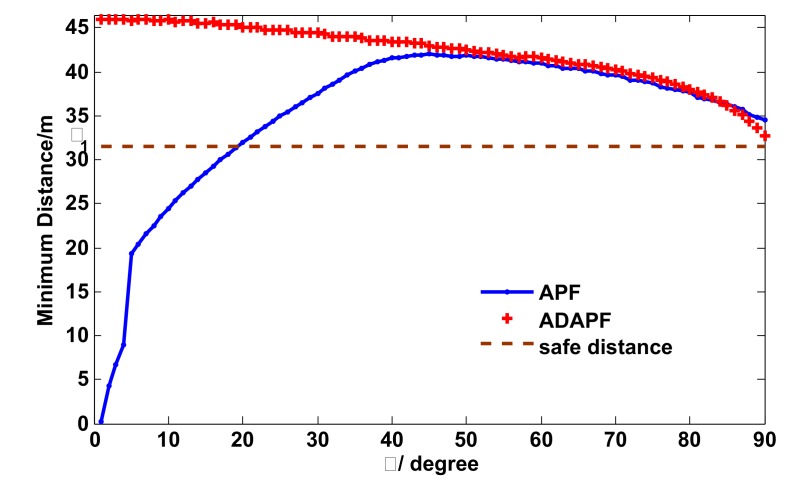
The angle vs. minimum distance.

As shown in Figure 6, the APF fails to guarantee that the MD is always larger than the minimum safety distance 31.5 m, while our path planner is reliable for all γ. Especially in the situation of $\gamma =0^{\circ }$ and $\gamma =1^{\circ }$, the agent using APF inevitably collides with the obstacle because the MD is zero.

### 5.3. Integrated Performance

**Figure 7:**
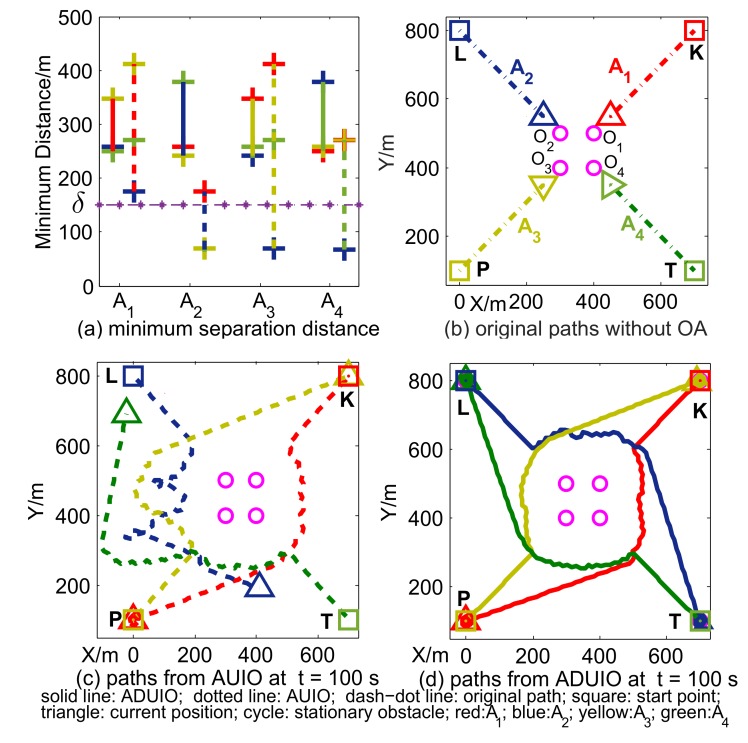
Minimum separation distances and trajectories of agents.

To test the integrated performance of obstacle estimation and angle-dependent APF, a scenario including agents $A_{1}-A_{4}$ and stationary obstacles $O_{1}-O_{4}$ was set up, as shown in Figure 7b. Every agent moves along a straight path with constant velocity without OA so that it will collide with two stationary obstacles and three other agents. Among scenario setups in Table 1, obs and pos are short for obstacle and position, respectively; CC obs represents the obstacle satisfying conditions of Theorem 1; and non-CC obs is the reverse.

Here, we compared between the combination of angle-dependent APF with UIO (ADUIO) and APF with UIO (AUIO). As shown in Figure 7a, minimum separation distances between each agent to other three moving agents by ADUIO are all above δ, while their AUIO counterparts are below δ. It is implied that paths generated by AUIO fail to avoid collision threats but succeed by ADUIO. As shown in Figure 2c,d, both methods avoid stationary obstacles successfully but cost different length of time to reach destinations. As shown in Table 2, the terminative time of ADUIO is much smaller than AUIO. In other words, our proposed scheme reaches the destination more quickly, while AUIO produces significant improper roundabout movements, as shown in Figure 7c, due to APF forces caused by CC obs. Results in Figure 7a,d also suggest our UIO guarantees estimation accuracy for the requirement of OA.

## 6. Conclusions and Future Work

This paper addresses the problem of automatic path planning using bearing-only measurements in the case that the obstacle motion is general. The reason for local minimum problem is discovered, based on which a variant of the APF is proposed to be the path planner. Then, an improved unknown input observer is proposed to estimate the positions and velocities of the obstacles. Finally, numeric simulations demonstrate the advantages of our method in terms of estimation accuracy and terminative time.

It needs to be noted that there are significant differences between our UIO and its traditional counterpart. First, our UIO avoids using the dynamic equations, which is necessary for the calculation in the traditional UIO. Second, our UIO is nonlinear, while traditional one needs to be linearized.

Nevertheless, our method only considers the case of the mass-point target and the accuracy is lacking theoretical proof. Therefore, our future research includes solving the problem about targets with different shapes, proving the convergence of the algorithms and considering the effects of noise. For example, one research line is about agents network techniques [26] involving clustering estimation, and another research line to be followed is to investigate the case where multiple agents are present and share their estimates simultaneously.

## Figures and Tables

**Table 1:** Scenario set up.

pos	***K***	***L***			***P***	***T***			$O_{1}$			$O_{2}$	$O_{3}$			$O_{4}$
	[700,800]		[0,800]		[700,800]		[700,800]		[700,800]		[700,800]		[700,800]		[700,800]	
plan				velocity				direction		CC obs				non-CC obs		
	$A_{1}$			[−10,−10]				K→P		$O_{1}$, $O_{3}$, $A_{3}$				$A_{2}$, $A_{4}$		
	$A_{2}$			[10,−10]				L→T		$O_{2}$, $O_{4}$, $A_{4}$				$A_{1}$, $A_{3}$		
	$A_{3}$			[10,10]				P→K		$O_{1}$, $O_{3}$, $A_{1}$				$A_{2}$, $A_{4}$		
	$A_{4}$			[−10,10]				T→L		$O_{2}$, $O_{4}$, $A_{2}$				$A_{1}$, $A_{3}$		

**Table 2:** Terminative time of two schemes.

	$A_{1}$	$A_{2}$	$A_{3}$	$A_{4}$
ADUIO/ AUIO	84 s/85 s	85 s/120 s	85 s/100 s	83 s/109 s
